# Granulocyte-macrophage colony stimulating factor (GM-CSF) is fully expressed in the genital tract, seminal plasma and spermatozoa of male pigs

**DOI:** 10.1038/s41598-020-70302-9

**Published:** 2020-08-07

**Authors:** Lorena Padilla, Jesús Martínez-Hernández, Isabel Barranco, Xiomara Lucas, Luis M. Pastor, Heriberto Rodriguez-Martínez, Jordi Roca, Inmaculada Parrilla

**Affiliations:** 1 Department of Medicine and Animal Surgery, Faculty of Veterinary Science, University of Murcia, 30100 Murcia, Spain; 2 Department of Cell Biology and Histology, School of Medicine, University of Murcia, 30100 Murcia, Spain; 3 IMIB-Arrixaca, Regional Campus of International Excellence, University of Murcia, Campus Mare Nostrum, 30100 Murcia, Spain; 4 Biotechnology of Animal and Human Reproduction (TechnoSperm), Department of Biology, Faculty of Sciences, Institute of Food and Agricultural Technology, University of Girona, 17003 Girona, Spain; 5 Department of Biomedical and Clinical Sciences (BKV), Linköping University, 58185 Linköping, Sweden

**Keywords:** Immunoblotting, Immunohistochemistry

## Abstract

Granulocyte-macrophage colony stimulating factor (GM-CSF) is a pro-inflammatory cytokine identified in boar seminal plasma (SP) but until now unexplored in terms of place of production and its association to spermatozoa. This study aimed to explore these aspects by evaluating the presence of GM-CSF in porcine reproductive organs (testes, epididymis and accessory sex glands), SP and mature spermatozoa (from cauda epididymis and ejaculated) using Western blot (WB), immunohistochemistry and immunocytochemistry. Positive labelling was obtained in tissues, SP and spermatozoa. In reproductive organs, WB revealed three forms of GM-CSF with different glycosylation degrees (15, 31 and 40 kDa). In SP and epididymal fluid, the GM-CSF appeared only in its active form while in spermatozoa the GM-CSF form present varied among sperm sources. Non-viable spermatozoa showed more GM-CSF than viable spermatozoa (14.87 ± 1.98 RU vs. 7.25 ± 0.52 RU) of fluorescence intensity. In conclusion, GM-CSF is widely present in the reproductive tract of male pigs, attached to the spermatozoa already in the epididymis as well as verted to SP. Consequently, the GM-CSF ought to regulate male genital tract and sperm function as well as mediating initial inflammatory responses and further mediating later immune actions by the female to semen deposition.

## Introduction

When insofar routinary semen analysis are hardly capable to identify sub-fertile sires^1,2^; finding effective biomarkers to predict the potential fertility of sires used in artificial insemination (AI) programs is a recurring challenge of the livestock industry; requiring alternative ways. One, and perhaps the most appropriate way to meet this challenge might be to explore the molecular composition of seminal plasma (SP) and of spermatozoa^3,4^. Indeed, some SP and sperm proteins have been postulated as suitable biomarkers of fertility^5–8^. Among semen proteins, the cytokines are recently acquiring notoriety because they would be directly involved in preventing pathogen colonization after semen deposition as well as the immune response of the female genital tract towards both spermatozoa, facilitating fertilization, and in embryos, facilitating their development and implantation^9,10^.

Pig semen is rich in cytokines^11^ and the role of some of them, particularly Transforming growth factors beta, on swine reproductive performance has deserved substantial scientific interest as they contribute to regulate the molecular reproductive events occurred in the female reproductive tract after natural or artificial mating, including the maternal immune response to embryos^12–16^. On the contrary, other semen cytokines, also present in high concentration in porcine SP^11^, have barely claimed scientific interest, for instance, the granulocyte and macrophage colony stimulating factor (GM-CSF). The GM-CSF is a pro-inflammatory cytokine with a molecular weight ranging from 50 to 15 kDa according to its glycosylation degree^17,18^. Immune regulatory functions as well as an active involvement on growth, proliferation, maturation, and function of hematopoietic cells have been described among the roles of this cytokine^19–21^. Currently, there are few studies that have evaluated the presence and distribution GM-CSF of the male reproductive tract. It has been described only in rat testes^22^ and its relevance in spermatogenesis has been merely hypothesized^23^. With this scenario in mind, the purpose of the present study was to improve our current knowledge of GM-CSF in reproduction, particularly its location and distribution alongside the male reproductive tract, SP and cauda epididymis and ejaculated spermatozoa, using immunohistochemistry (IHC), immunocytochemistry (ICC) and Western blot (WB) procedures.

## Results

### GM-CSF is immunohistochemically detected in pig testes, epididymides and accessory sex glands

**Figure 1:**
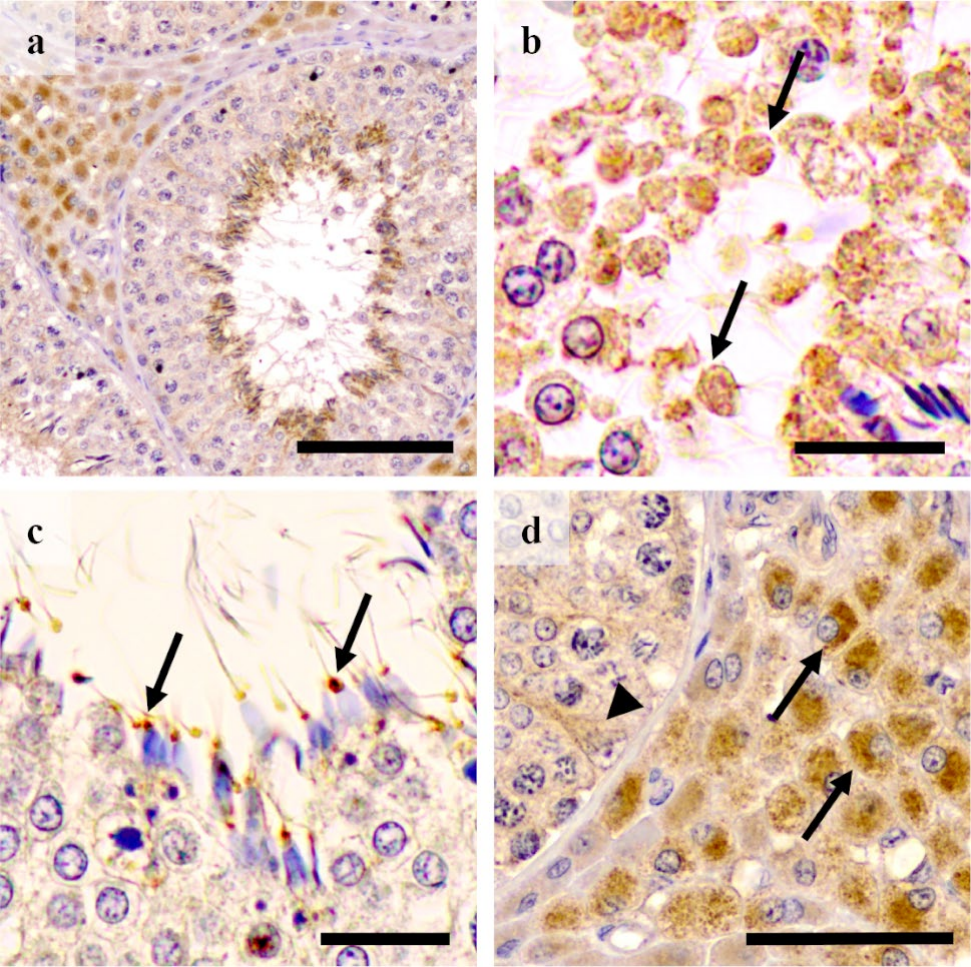
Immunohistochemistry of GM-CSF in pig testes. Positive immunolabelling is seen in (**a**) the interstitium (Leydig cells) and the residual bodies of elongated spermatids. (**b**) Detail of positivity in residual cytoplasm derived from spermatids (arrows). (**c**) Expression in cytoplasmic droplets attached to neck region of immature spermatozoa (arrow). (**d**) Strong cytoplasmic immunostaining in Leydig cells (arrows) and weak in Sertoli cells (arrowhead). Scale bars: (**a**) 100 μm; (**b** and **c**) 20 μm; and (**d**) 50 μm.

**Figure 2:**
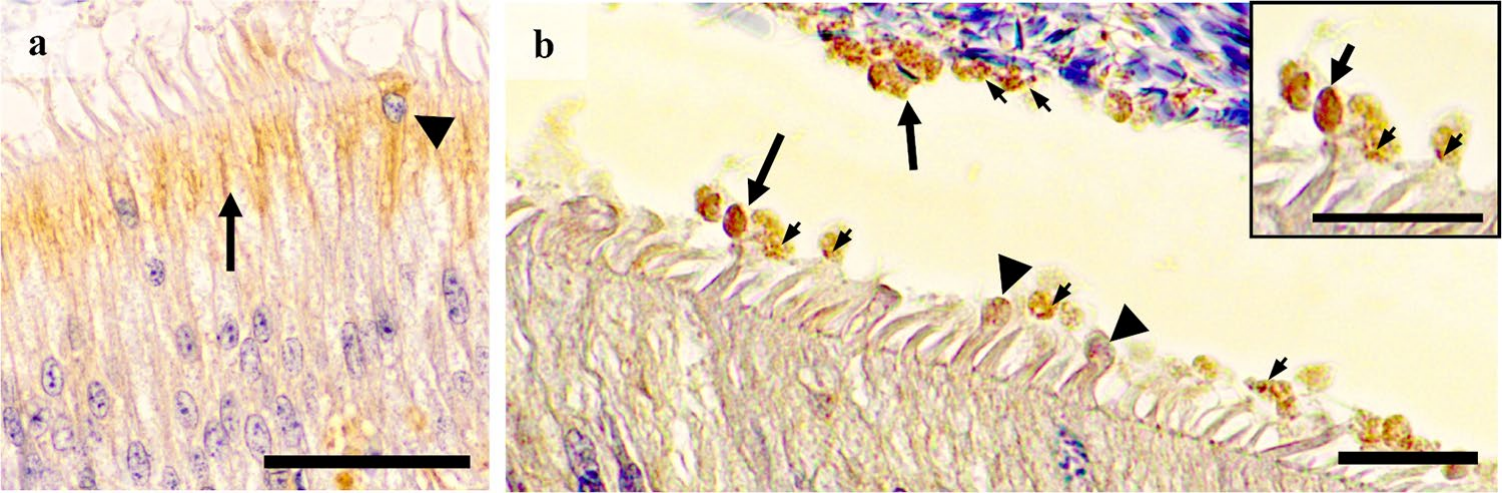
Immunohistochemistry of GM-CSF in pig epididymis. Immunoreactivity in (**a**) supranuclear region of the principal cells (arrow) of the epithelium from the caput segment. Arrowheads identifies apically-stained cells, and intralumenal vesicles positive (**b**) to GM-CSF (the relative size of the arrows used illustrate differences in vesicle size and arrowheads mark apical protuberances of principal cells). Insert: gross arrow: positive vesicle; narrow arrows: smaller positive vesicles inside the vesicles. Scale bars: (**a**) 50 μm; and (**b** detail) 20 μm**.**

**Figure 3:**
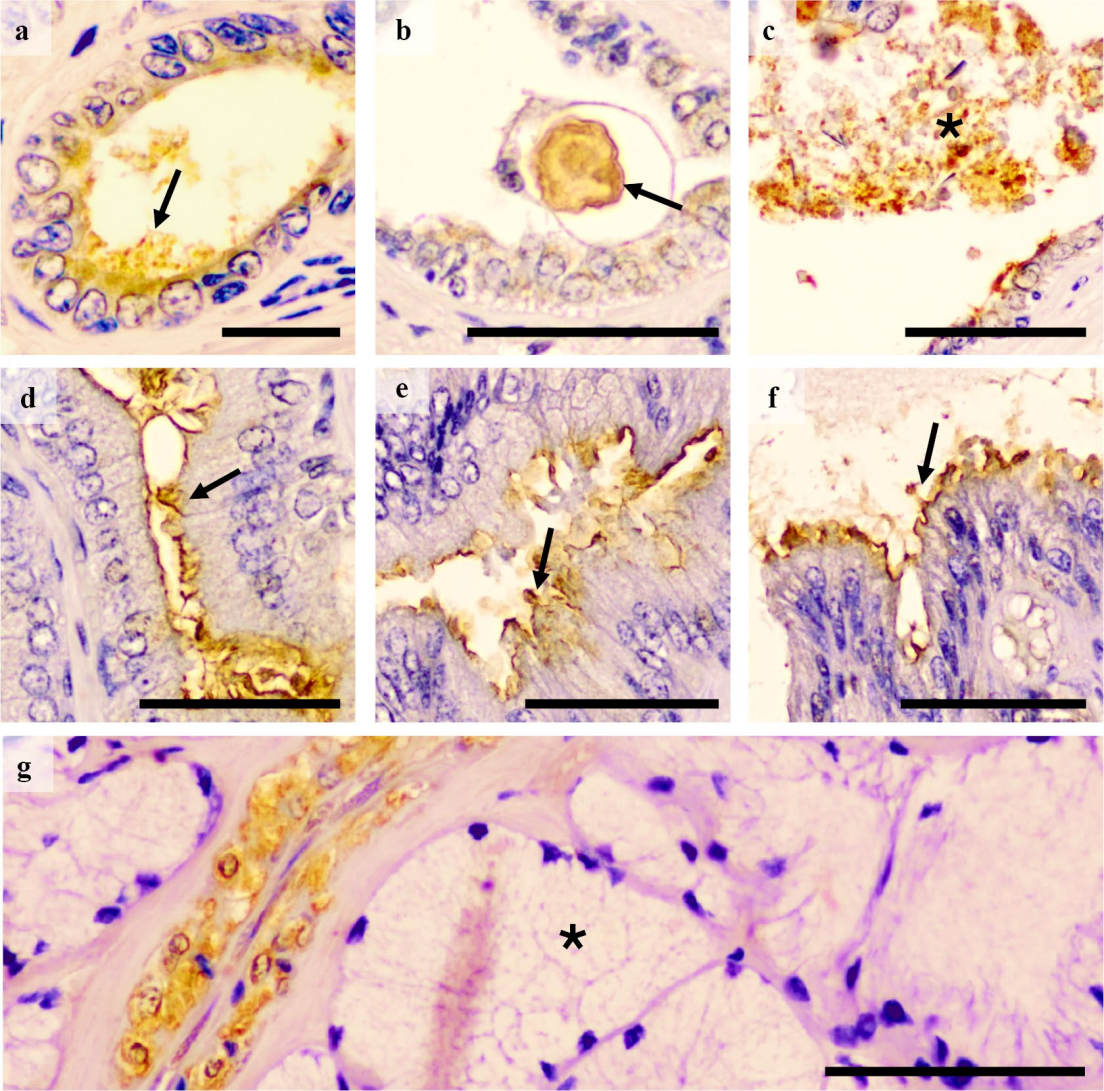
Immunohistochemistry of GM-CSF in pig accessory sex glands. Immunolabelling in (**a**) prostate, present in supranuclear cytoplasm and small vesicles (arrow) in the lumen; (**b**) corpora amylacea (arrow); (**c**) vesicles in lumen (asterisk); (**d**–**f**) Seminal vesicle with staining in protruding apical membranes (arrows) and (**g**) the bulbourethral gland where immunostaining was observed in vascular smooth muscle, but not in secretory epithelium (asterisk). Scale bars: (**a**) 20 μm; and (**b**–**g**) 50 μm.

All reproductive tissue samples expressed GM-CSF. In the testes, the GM-CSF was present both in the seminiferous epithelium and in the interstitium. In the seminiferous epithelium, it was observed in residual cytoplasm derived from spermatids, in the neck of elongated spermatids and also, albeit weakly, in Sertoli cells (Fig. 1a–c). In the interstitium, GM-CSF was expressed in Leydig cells, particularly in their cytosol (Fig. 1d). GM-CSF in the epididymis was present in the supranuclear region of the principal cells of the epididymal epithelium, markedly in the corpus epididymis, and in clear cells along the epididymis (Fig. 2a). In addition, the apical blebs of epididymis epithelial cells also showed GM-CSF, being the presence more notable when these vesicular structures were freed in the lumen. The epididymal spermatozoa displayed GM-CSF in their proximal cytoplasmic droplet as well as in small luminal vesicles (Fig. 2b). Relative to the accessory sex glands; the prostate showed GM-CSF in the supranuclear cytoplasm and in the apical edge of both the principal and secretory epithelial cells (Fig. 3a); and in intralumenal secretory vesicles (Fig. 3b). Occasionally, prostate corpora amylacea also showed GM-CSF (Fig. 3c). The seminal vesicle showed GM-CSF in the glandular epithelium, more specifically in the apical membrane and membrane protuberances of the secretory cells (Fig. 3d–f). Finally, the GM-CSF in the bulbourethral glands was exclusively present in the smooth muscle surrounding the arterioles (Fig. 3g).

### GM-CSF is expressed in epididymal and ejaculated pig spermatozoa

**Figure 4:**
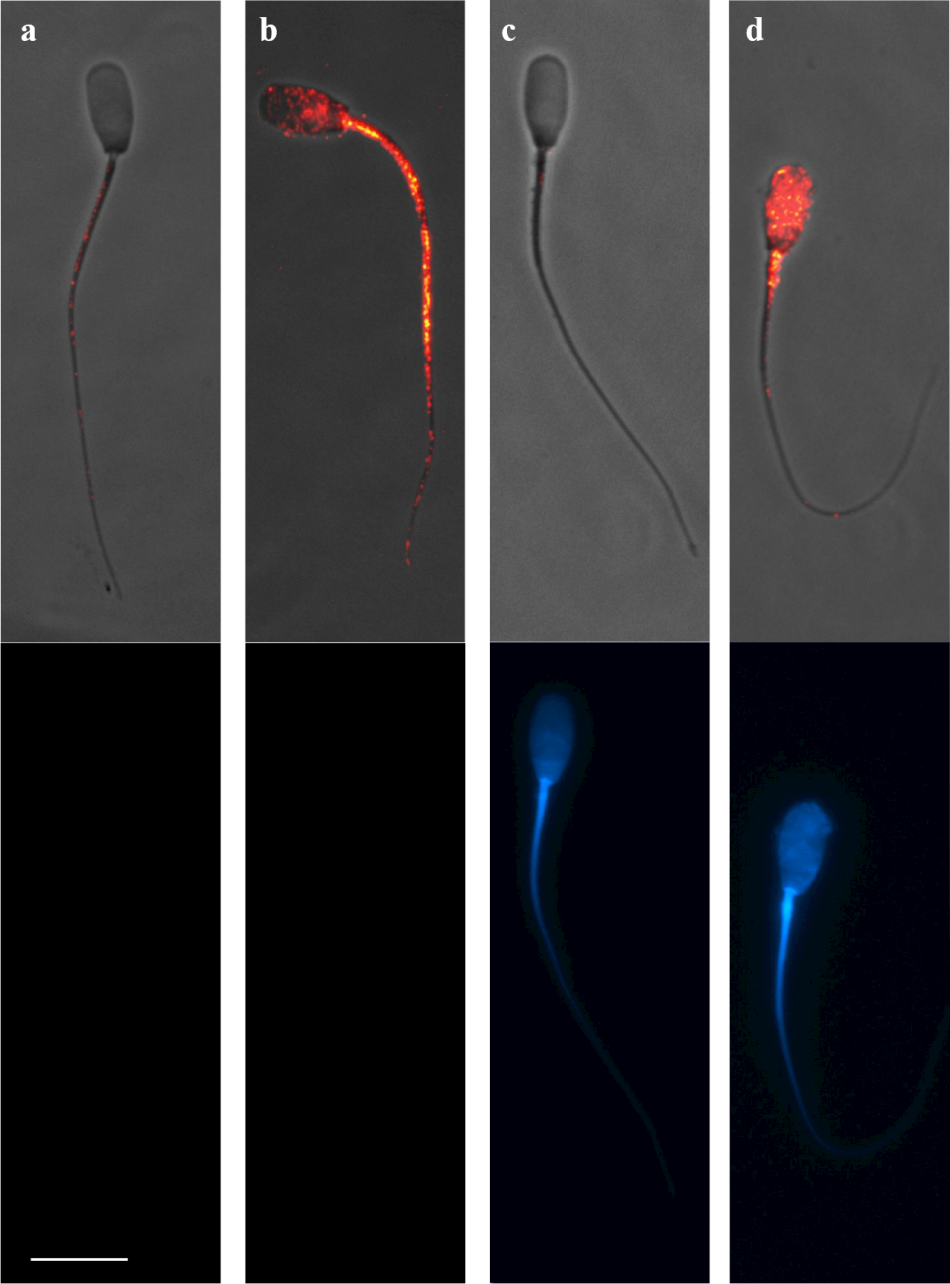
Immunocytochemistry of GM-CSF in the pig mature spermatozoa. Top images (**a** and **c**) show spermatozoa with low/absent GM-CSF expression and bottom images show spermatozoa viable and non-viable (DAPI stain). The sperm samples were incubated with anti-GM-CSF antibody (orb6090, Biorbyt) to identify GM-CSF expression (composite in red in top images) and incubated with DAPI staining to identify viable from non-viable (DAPI positive in blue in bottom images) spermatozoa. Sperm in (**a**) and (**c**), were included in cluster 1 (fluorescence intensity 4.45 ± 0.17 RU; ranging from 0.0 to 15.33 RU) while spermatozoa displaying high GM-CSF expression were included in cluster 2 (fluorescence intensity 29.81 ± 1.82 RU; ranging from 15.67 to 84.0 RU, **b** and **d**). Scale bar: 10 μm. Cropped image of immunocytochemistry capture (see Supplemental Information, Fig S5 for full image).

**Figure 5:**
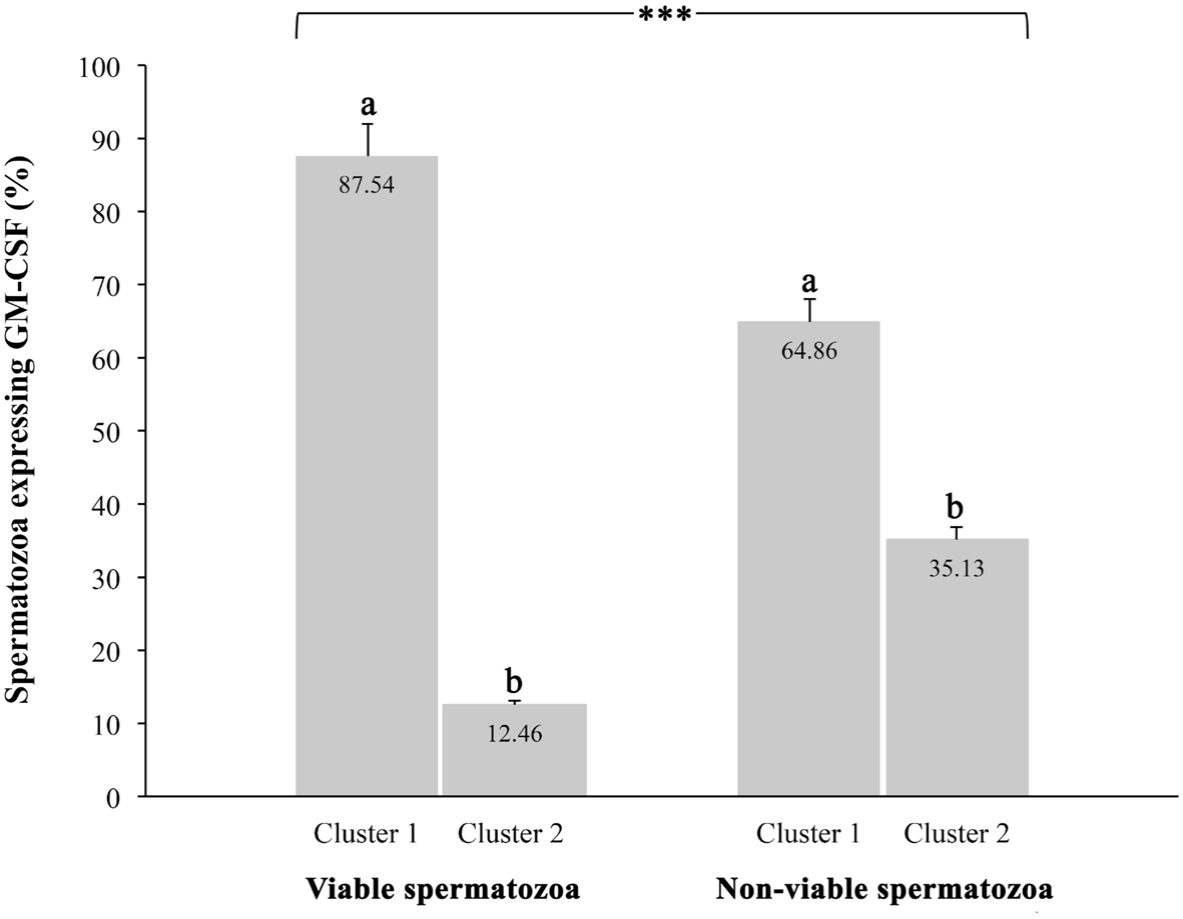
Proportion of spermatozoa hierarchically clustered in two populations according to the expression of GM-CSF in the viable and nonviable sperm population. ^a,b^ Indicate significant differences (*p* < 0.01) between cluster 1 and 2. The cluster 1 gathered the spermatozoa showing low/absent fluorescence intensity (4.45 ± 0.17 RU; ranging from 0.0 to 15.33 RU) corresponding with a low/absent GM-CSF expression. The cluster 2 gathered those sperm displaying high fluorescence intensities (29.81 ± 1.82 RU; ranging from 15.67 to 84.0 RU).

Spermatozoa were hierarchically clustered (*p* < 0.01) into two groups according to the fluorescence intensity displayed, which was directly related to GM-CSF relative amounts. The first cluster (cluster 1) gathered the spermatozoa showing low/absent fluorescence intensity (4.45 ± 0.17 RU; ranging from 0.0 to 15.33 RU) corresponding with a low/absent GM-CSF expression. The second cluster (cluster 2) gathered those sperm displaying high fluorescence intensities (29.81 ± 1.82 RU; ranging from 15.67 to 84.0 RU) (Fig. 4) corresponding with a high presence of GM-CSF. The proportion of spermatozoa in each cluster for viable and non-viable spermatozoa is showed in Fig. 5. The number of spermatozoa in cluster 1 (with low/absent presence of GM-CSF) was higher (*p* < 0.05) than in cluster 2 (high presence of GM-CSF), irrespective of they were viable or non-viable. However, the fluorescence intensity was higher (*p* < 0.01) in non-viable than in viable spermatozoa (14.87 ± 1.98 RU vs. 7.25 ± 0.52 RU, respectively). Looking at fluorescent spermatozoa, they displayed a heterogeneous distribution in fluorescence signal, but it was usually more intense in midpiece and sperm flagellum, which was particularly evident in non-viable spermatozoa (Supplementary Figure S1).

### Western blot confirmed the presence of GM-CSF in reproductive organs and derived fluids and in mature pig spermatozoa

**Figure 6:**
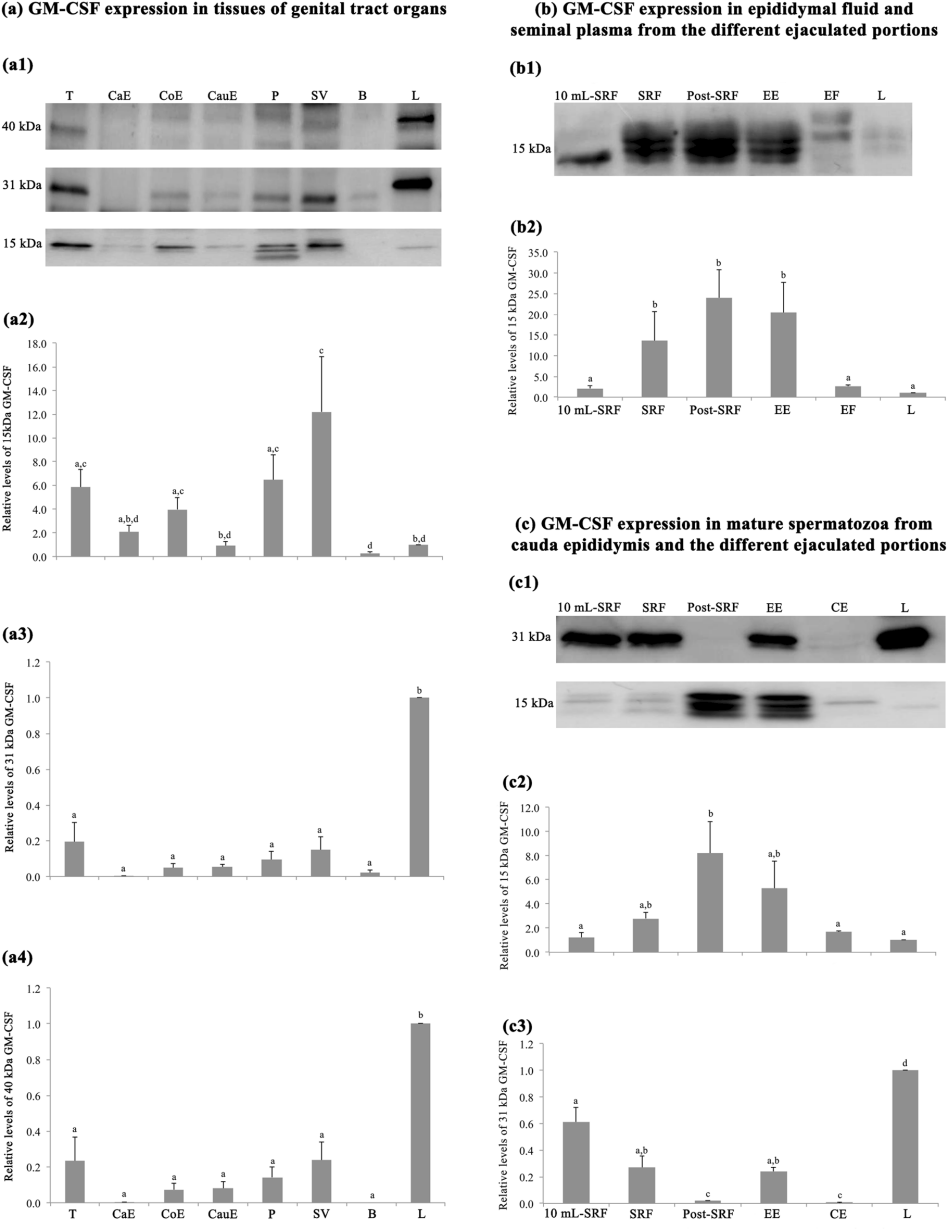
Western blot analysis and relative quantification of GM-CSF expression in porcine genital tract, epididymal fluid (EF), seminal plasma (SP) and in spermatozoa from cauda epididymis (CE) and different ejaculate portions. In (**a1**), three bands of GM-CSF expression with the different glycosylation degrees: 15, 31 and 40 kDa. In (**a2**–**a4**), the relative quantity of 15 kDa (**a2**), 31 kDa (**a3**) and 40 kDa (**a4**) in reproductive tissues; T: testes. CaE: caput epididymis. CoE: corpus epididymis. CauE: cauda epididymis. P: prostate. SV: seminal vesicle. B: bulbourethral gland. In **(b1**) one band of active GM-CSF expression of 15 kDa. In (**b2**) relative quantity of 15 kDa in EF and SP from the different ejaculate portions: first 10 mL-SRF, rest of SRF, post-SRF, entire ejaculate (EE). (**c1**) Two bands of active and glycosylated GM-CSF of 15 and 31 kDa. (**c2** and **c3**) Relative quantity of 15 kDa (**c2**) and 31 kDa (**c3**) in mature spermatozoa from CE, first 10 mL-SRF, rest of SRF, post-SRF and EE. L: expression in liver tissue, as control. ^a–d^ Indicate relative quantitative differences (*p* < 0.05) among tissues, fluid and sperm sources. SRF: sperm-rich fraction. Cropped image of Western blot (see Supplemental Information, Fig S6 for full image).

Western blot identified three different forms of GM-CSF in the pig reproductive tissues, with molecular weights of ~ 40, 31 and 15 kDa, see Fig. 6(a1), like to those identified in positive control of liver tissue. The 15 kDa form was the only one with different relative amounts (*p* < 0.05) among the reproductive tissues. The testis, corpus epididymis, prostate and seminal vesicle glands had higher amounts than the cauda epididymis and bulbourethral glands, see Fig. 6(a2). There was no measurable amount of the 31 kDa form in the caput epididymis (Fig. 6,a3) whereas that of 40 kDa form was neither detected in the caput region nor the bulbourethral glands, see Fig. 6(a4).

In contrast to the tissues, only the 15 kDa form was identified in the fluids, either in the SP or in the cauda epididymal fluid, see Fig. 6(b1). The epididymal fluid and the ejaculate revealed fraction differences; epididymal fluid and the SP from the first 10 mL of the sperm-rich fraction (SRF) had significantly (*p* < 0.05) lower amounts of this GM-CSF form, compared to the rest of the SRF, post-SRF and entire ejaculate (EE), see Fig. 6(b2). Mature spermatozoa retrieved from all sources, namely from the first 10 mL-SRF, the rest of SRF, post-SRF, the EE and the cauda epididymis, showed the 15 and 31 kDa GM-CSF forms, see Fig. 6(c1). Differences in relative amount among the sperm sources were identified for both GM-CSF forms (*p* < 0.05). The relative amount of 15 kDa form was greater in spermatozoa from the post-SRF than in those of the first 10 mL-SRF, rest of SRF and cauda epididymis, see Fig. 6(c2). In contrast, the 31 kDa form was present in smaller quantity in spermatozoa from the post-SRF and in those of cauda epididymis Fig. 6(c3).

## Discussion

To the best of our knowledge, this is the first study demonstrating that reproductive tissues, fluids and gametes (immature and mature spermatozoa) of male pigs express GM-CSF. In addition, the WB demonstrated that GM-CSF was expressed both in active form with low/poor glycosylation, with a molecular weight of 15 kDa, and in highly glycosylated forms, with molecular weights of 31 and 40 kDa. Higher glycosylated forms have an extended half-life in the body fluids in contrast to the active form that has a very short functional half-life^20,24^. The WB performed with tissue samples also revealed the presence of two non-specific bands (25 and 50 kDa) in liver and in the rest of tissues. These non-specific bands were detected by the secondary antibody, belonging to the light (25 kDa) and heavy (50 kDa) chains of the endogenous immunoglobulins of the animal tissue itself (porcine IgG)^25^. This finding was confirmed when the WB was performed with the primary antibody absent.

The IHC demonstrated that GM-CSF was widely present in the reproductive tissues (organs) of male pigs. The testis showed both lower and higher glycosylated forms, but the latter apparently in low quantity as revealed by WB densitometry. Since the functional half-life of active GM-CSF form is very short^20^, it is reasonable to consider that the active GM-CSF with low/poor glycosylation, present in the testes would play its functional role in the testes themselves. Probably, this active form would be the one present in functional cells, particularly in Leydig compared to Sertoli cells, perhaps contributing as a spermatogenesis-controlling factor^26^. The presence of the active GM-CSF form in Leydig cells could be associated with the close morphological and functional relationship of Leydig cells with testicular macrophages (reviewed by Hutson^27^), the latter having a high GM-CSF synthesis capacity^22,28^. This finding, together with the presence of GM-CSF in the cytoplasmic droplets remaining attached to the neck region of testicular spermatozoa, would indicate that this cytokine would be involved in pig spermiogenesis, or perhaps even in spermatogenesis, as suggested by Dirami et al*.*^23^ who showed that GM-CSF influenced positively or negatively pig spermatogonial survival in vitro, in a concentration-dependent manner.

The epididymis also showed both active and higher glycosylated forms of GM-CSF, but maintaining the same WB densitometry picture, with the active form dominating, being conspicuous in caput and corpus compared to cauda epididymides. GM-CSF stimulates the proliferation and maturation of granulocytes and macrophages, cells present in the epithelium and epididymal interstitial tissue and even passing to the epididymal lumen^29^ probably to maintain an appropriate immunological environment for spermatozoa and also for protecting them from potential pathogens^30^. The distribution of GM-CSF we documented, follows the distribution seen for macrophages in rodents^30–32^, suggesting there might also be a putative greater presence of macrophages in these epididymal regions in boars, a matter we have not specifically mapped in the present study. A matter to consider, is that our relative distribution of both active form (low glycosylation) and highly glycosylated forms of epididymal GM-CSF, considered hereby the contribution of vesicles and residual cytoplasmic bodies^33^.

The GM-CSF was also present in the accessory sex glands, mainly in prostate and seminal vesicles, mostly present as poorly glycosylated (active) in both glands. This would indicate that these glands have the ability to synthesize GM-CSF, which would be released in the luminal compartment, mainly as active form, and thus become a component of the SP, as hereby demonstrated by WB^11^. The relatively high expression of active GM-CSF (15 kDa) found in most of the boar tissues analysed in comparison with the control liver tissue, could be related with a distinct functional state of the cytokine. It has been recently described that in liver an increase in this cytokine is produced by inflammation and fibrosis processes^34^. The tissue samples used in our study come from a healthy liver; which could be a reasonable explanation for the low expression of the detected 15 kDa GM-CSF form.

The WB also revealed that the active GM-CSF was in greater quantity in the SP of particular ejaculate fractions, namely the so-called “rest of the SRF” and the post-SRF, just the two pig ejaculate portions gathering most of the secretion from prostate and seminal vesicles^35^. The GM-CSF is a key regulator of the immune and inflammatory response^36^ and its presence in SP would be involved in the positive effects exerted by SP on the uterus of sows for promoting an initial, transient inflammation, probably mostly directed to eventual pathogens; but also influencing both sperm survival and embryo development^13,37^. In this context, it is well known that GM-CSF is a cytokine that improves embryo development and the establishment of pregnancy in rodents^38^.

Interestingly, our study showed for the first time the presence of GM-CSF in mature spermatozoa. Certainly, mature spermatozoa from both the cauda epididymis and the ejaculate showed both lower and higher glycosylated GM-CSF forms, particularly in the sperm tail, both in midpiece and the rest of the tail. Mature spermatozoa are considered transcriptional and transnationally silent^39^, thus lacking capacity to synthesize cytokines. Then, it would be reasonable to believe that GM-CSF present in mature spermatozoa without a retained cytoplasmic drop, is attached from that GM-CSF in the lumen of the epididymis and/or the SP, as it happens with many other adhering SP proteins^40^. This statement would be entirely convincing to explain the overwhelming presence of the active GM-CSF with low glycosylation since it is the one widely present in SP, particularly in the SP from post-SRF. Moreover, the distribution of the active GM-CSF in spermatozoa and SP derived from the different ejaculate portions was very similar, thus supporting the above claim. This active GM-CSF present in mature spermatozoa would exert a regulatory activity in the cell. Studies in human, bovine and ovine species described the existence of GM-CSF receptors in the midpiece and principal segment of the tail of mature spermatozoa^41–43^ and also demonstrated that GM-CSF was able to improve motility when added to bovine sperm samples^41^. However, the above theory about the bind of GM-CSF from SP to mature spermatozoa would not explain the capability of mature spermatozoa to express the glycosylated form of 31 kDa, as it was not present in SP. Two possible explanations ought to be considered. Firstly, the glycosylated form of GM-CSF would be a native protein of spermatozoa derived from spermatogenesis as demonstrated by its presence in the residual bodies released from spermatids during final spermiogenesis. The low relative abundance of this cytokine in spermatozoa would explain why it was not identified in the current pig sperm proteome^44,45^, maybe due to the lack of sensitivity of the current methods for proteomics failing to identify low abundant peptides, as it would be the situation for many cytokines^5^. Secondly, this glycosylated form could be inside the SP-extracellular vesicles that are attached to the spermatozoa during ejaculation. It is well known that virtually all epithelia lining the male genital organs, including those of epididymis and accessory sex glands, have the capability to generate and release extracellular vesicles^46^. Extracellular vesicles play a key role in cell-to-cell communication and they transport many functional molecules, including cytokines, from donor to target cell^47,48^. Extracellular vesicles have the ability to bind to spermatozoa during its transit along the male genital tract, transferring its cargo to spermatozoa, as demonstrated in vivo in bovine^49^ and also in vitro in pigs^50^.

Another interesting result was that the GM-CSF was more present, in the non-viable than viable spermatozoa, as demonstrated by differences in fluorescent intensity. A plausible explanation for this finding could be that the membrane damage characterizing non-viable spermatozoa would facilitate the entry of the anti-GM-CSF antibody inside the spermatozoa, binding to the existing inner GM-CSF. In this regard, we previously demonstrated that GM-CSF was indeed particularly evident in frozen-thawed—often presenting high proportions of membrane-damaged spermatozoa^51^—compared to fresh boar semen^52^. Contrary, those viable sperm (with an intact plasma membrane) did not allow the entry of antibody, leading to low fluorescence intensity in the majority of viable spermatozoa and determining the high proportion of viable spermatozoa in cluster 1 (87.54%). On other hand, the low percentage of viable spermatozoa showing high expression of GM-CSF (12.46%) could be, most likely, related to sperm displaying apoptotic-like changes that results in a slight increase of cytoplasmatic membrane permeability allowing the antibody entrance^53^. This finding would support that boar spermatozoa have intracellular GM-CSF, which would be released once in the sow genital tract when spermatozoa experienced membrane destabilization^54^.

In conclusion, the results of the present study demonstrate for the first time that GM-CSF, both in poorly glycosylated (active) and highly glycosylated form, is present in the genital tract of the male pig, and that it is transported to the SP and in spermatozoa. The active form is solely present in the cauda epididymal fluid and in the SP, regardless of the fraction of the ejaculate considered. On the other hand, the form of GM-CSF found in spermatozoa is influenced by the origin of the sperm sample. The relevance that these findings could have on the reproductive physiology of male and female pigs has not yet been elucidated. Further studies are required to improve the knowledge about the exact role of GM-CSF in regulating the cellular and molecular mechanisms of the immune system that underlie the successful establishment of pregnancy in pigs.

## Material and methods

### Reagents and media

All chemicals used in the experiments were for analytical purpose. All media components and fluorochrome molecules, unless otherwise indicated, were purchased from Merck KgaA (Darmstadt, Germany, Europe), and the media were prepared under sterile conditions. The basic media used to dilute reagents, antibodies and fluorochrome molecules was EDTA-free phosphate-buffered saline (PBS: NaCl 138 mM, KCl 2.7 mM, KH_2_PO_4_ 1.5 mM, Na_2_HPO_4_·7H_2_O 8.1 mM; pH 7.4 ± 0.2; 288 ± 8 mOsmol/kg, at 25 °C). The washing between incubations in IHC and WB protocols were performed using Tris-buffered saline (TBS: NaCl 1,500 mM, Tris Base 200 mM, HCl 12 N; pH 7.6, at 25 °C). The loading buffer use in WB was made in our laboratory using 125 mM Tris-HCl (Tris Hydrochloride for buffer solutions, PanReac AppliChem ITW Reagents, Barcelona, Spain), glycerol at 10% (Glycerol 99%) SDS at 2%, 2-mercaptoetanol at 5% and bromophenol blue at 0.01% (Fluka Analytical, Honeywell Fluka™, Morris Plains, NJ, USA).The semen extender used was Beltsville Thawing Solution (BTS;^55^).

### Animals

All procedures that involved animals were performed following international guidelines (Directive 2010/63/EU) and were approved in advance by the Bioethics Committee of Murcia University (research code: 639/2012).

Healthy, fertile and mature boars of different breeds (Large White, Landrace and Duroc), trained to be collected from a dummy and undergoing regular semen collections (two ejaculates collected per week with a 3 days interval between collections) in a commercial AI center (AIM Ibérica, Calasparra, Murcia, Spain) were used. Boars were housed in individual pens under environmentally-controlled conditions (15–25 °C; 16 h of natural/artificial daylight) having free access to water and fed with a commercial feedstuff that met the nutritional requirements for adult boars used for regular ejaculate collection^56^.

### Ejaculate collection and processing

Only ejaculates meeting the quality standard limits for the preparation of semen AI-doses were used in this study (Supplementary Table S1). Ejaculates were collected using the gloved-hand method, allowing separate collection of three different ejaculate portions as follows: the first 10 mL-SRF (sperm-peak portion), the remaining SRF and the post-SRF. These 3 portions correspond with the 3 analysed samples. A fourth sample, mimicking the EE, was generated by the proportional mixture of the other three portions 10 mL-SRF, rest of SRF and post-SRF as described previously by Perez-Patiño et al*.*^45^. Each one of four samples were separately centrifuged at 800 × *g* for 8 min (Eba 20, Hettich Zentrifugen, Germany) immediately after collection to separate spermatozoa from the SP. Both, spermatozoa and SP samples were placed in sterile tubes and transported in cooled box (4 °C) to the Veterinary Andrology Laboratory of the University of Murcia. Once in the laboratory, each sperm sample was split into two aliquots and whereas one was stored at − 80 °C until use for WB analysis, the other was immediately processed for ICC as described below. The SP samples were twice centrifuged at 1,500 × *g* for 10 min (Sorvall ST 40R Centrifuge, Thermo Scientific, MA, USA) to remove any remaining spermatozoa or cell debris and, then, were stored at − 80 °C until use for WB analysis.

### Tissue sample collection

The boars, still healthy and fertile, were slaughtered following commercial decision based on genetic progress and replacement reasons, at a commercial slaughterhouse (MercaMurcia; Murcia; Spain). The reproductive organs, specifically the testes and sexual accessory glands, were collected immediately after slaughter and dried with sterile cloths to remove the remains of blood. Thereafter, tissue sections of 1.5 × 1.5 cm of testis, epididymides (caput, corpus and cauda epididymis), prostate, seminal vesicle and bulbourethral glands were retrieved for IHC analysis. The tissue sections were immersed into 30 mL of Bouin solution at room temperature (RT) and transported to the Veterinary Andrology Laboratory of the University of Murcia. Once at the laboratory and after 12 h of fixation, tissue samples were immersed in alcohol 70% to remove picric acid, and then embedded in paraffin blocks, sliced and mounted on slides. Tissue sections, of 0.5 × 0.5 cm, of the same reproductive organs were frozen into cryotubes by plunging them in liquid nitrogen vapours and thereafter stored at − 80 °C until WB analysis.

### Cauda epididymal spermatozoa collection and processing

The cauda epididymal content (spermatozoa and fluid) were retrieved at the laboratory by aspiration from the proximal end of the cauda epididymis after retrograde infusion of air through the ductus deferens. Once retrieved, the cauda epididymal-content samples were centrifuged at 800 × *g* for 8 min (Sorvall™ Legend Micro 21 R Centrifuge, Thermo Scientific) and the resulting sperm pellets processed for ICC as described below. The supernatant (epididymal fluid) was handled just like SP, for WB analysis.

### Sperm immunocytochemistry (ICC)

Firstly, sperm samples (30 × 10^6^ spermatozoa in 1 ml of BTS) were incubated (37 °C for 15 min) with 50 μL (50 µg/mL in PBS) of DAPI (4′,6-diamidino-2-phenylindole) to discern viable from non-viable spermatozoa. Then, sperm samples were centrifuged at 800 × *g* for 8 min at RT and fixed in 1 mL of 4% paraformaldehyde (v/v; 32% paraformaldehyde aqueous solution, Electron Microscopy Sciences, Hatfield, PA, USA in PBS). The fixed samples were again centrifuged, and the resulting sperm pellets re-extended in PBS to prepare smears with 25 µL/sample on poly-L-lysine slides. The smears were then blocked with PBS-Glycine 0.02 M at RT for 20 min, washed (2 times in PBS for 5 min) and incubated with the primary antibody against GM-CSF [1:200 in PBS 0.1% BSA (v/w); GM-CSF polyclonal rabbit orb6090, Biorbyt, St. Francisco, CA, USA] at 4 °C, overnight. Thereafter, the smears were washed and incubated, with the secondary antibody (1:200 in PBS 0.1% BSA; Goat Anti-Rabbit IgG Antibody, biotin-SP conjugate), at RT in the darkness for 60 min; before being further washed and incubated with Streptavidin (1:400 in PBS 0.1% BSA, Streptavidin, Alexa Fluor TM 555 conjugate, Thermo Fisher Scientific, Barcelona, Spain), at RT in darkness for 20 min. Finally, the smears were again washed and mounted with 2.5 μL of Vectashield antifade mounting medium (Vector Laboratories, CA, USA). Smears without the primary antibody were used as negative controls. The smears were examined in a Nikon Eclipse E800 fluorescence microscope (Nikon Instruments Corporation, Melville, New York, USA) equipped with DS-QiMc Nikon camera and a total of 200 spermatozoa were evaluated per sample. The spermatozoa were localized in bright-field and then evaluated by fluorescence microscopy for DAPI and Streptavidin Alexa Fluor, using filters for blue and red fluorescence (DAPI: 416 nm and 555: 565 nm, respectively) at 400 × to 1,000 × magnification range. The total intensity of GM-CSF fluorescence was calculated at a 16-bit grayscale level in regions of interest rounding each spermatozoon. The grey intensities were analysed with Image J software^57^, measured on a scale of 0 (black) to 255 (white) and expressed as relative units (RU).

### Immunohistochemistry (IHC)

The immunolabelling of GM-CSF in genital tract tissues was done using the avidin-biotin-complex method and following the recommendation of the manufacturer (Vector Laboratories, Burlingame, CA, USA). Briefly, 4 µm thick section of each tissue was dewaxed and progressively rehydrated from 100 to 50% ethanol and placed in distilled water. All washings between incubations was performed with 5 mM TBS at pH 7.6 (3 times for 5 min at RT). A previous antigen retrieval step was performed before IHC by placing the slides with the tissue into citrate buffer pH 6.1 (Dako, EnVision™, Denmark) solution inside a pressure cooker during 30 min at 98 °C; followed by a wash of slides during 30 min with a blocking solution (100 µL per slide; 3% [v/v] H_2_O_2_ in methanol) for endogenous peroxidase inactivation. Then, as the first step of IHC procedure, the slides were incubated with the same primary antibody against GM-CSF used in ICC (4 °C, overnight). After proper washing, slides were incubated with a polyclonal goat anti-rabbit secondary antibody conjugated with biotin (AP132B; Goat Anti-Rabbit IgG Antibody, biotin-SP conjugate, Merck, Germany) diluted in 1:200 in PBS supplemented with 0.1% of BSA (RT, 30 min). Slides were then incubated with Avidin-Biotin complex using the Vectastain Elite ABC kit for 1 h, and labelling was performed using peroxidase-based staining with 3,3′-diaminobenzidine (DAB, Vector Labs, Burlingame, USA). Slides were counterstained with Mayer’s haematoxylin, dehydrated, mounted and microscopically (Olympus BX51, Olympus DP25 camera) evaluated using 400 × , 600 × and 1,000 × range magnification. Positive (incubation with GM-CSF [1 mg/mL]) and negative (non-incubation with primary antibody) controls were performed on contemporary slides of pig liver tissue (Supplementary Figure S2).

### Western blotting (WB)

Genital tract, spermatozoa, SP and epididymal fluid samples were thawed in ice at 4 °C and proteins were extracted by homogenization and incubation in ice (4 °C) with extraction buffer PBS-SDS at 1% (Merck, Schuchardt, Germany) supplemented with protease inhibitors (Complete Mini EDTA-free; Roche, Mannhein, Germany) for 60 min. Then, protein samples were centrifuged 15,000 × *g* at 4 °C for 45 min and the supernatants collected. Protein quantification was done using the BCA protein Assay Kit (Thermo scientific, Rockfordd, USA). The precipitation of proteins was performed with acetone in order to eliminate the effect of interfering substances. The protein suspension was denatured in loading buffer by heating them to 95 °C for 5 min and was loaded (20–30 μg) per lane into a Mini-PROTEAN TGX precast gels 4–15% Bis-Tris SDS-PAGE gel (Bio-Rad Laboratories, USA). Electrophoresis was performed with protein standards Precision Plus Protein Dual Colour Standards (Bio-Rad Laboratories, USA), run at 180 V for 40 min. The proteins were transferred to an Immobilon-P membrane (Millipore, Bedford, MA, USA) by semidry electrophoretic transfer at 240 mA, for 60 min. Once the proteins were transferred to the membrane, the membranes were kept in TBS-Tween-20 0.2% supplemented with 5% dry milk non-fat (TBS-Tm), at 4 °C overnight. Thereafter, the membranes were washed three times (5 min each) in TBS-Tm and incubated in the primary rabbit polyclonal antibody against GM-CSF (Biorbyt, St. Francisco, CA, USA) (dilution 1:500 in TBS-Tm) at 4 °C, overnight. Following three washes in TBS-Tm, incubation with the secondary antibody goat anti-rabbit IgG biotinylated (Millipore, Temecula, California) (dilution 1/3,500 in TBS-Tm) followed during 90 min with a final wash in TBS-Tm. Subsequently, the membranes were incubated with Horseradish peroxidase (HRP) Streptavidin (GE Healthcare, UK) diluted 1/3,500 in TBS-Tm for 45 min. Immunoreactive bands were located with Clarity Western ECL Substrate (Bio-Rad Laboratories, USA). The images of the blotting were obtained using the ImageQuant LAS 500 (Healthcare Life Sciences) and densitometry was used with Image J software^57^. Ponceau was used for total protein normalization^58^. Pig liver, used as positive control, expressed GM-CSF in three different forms, with molecular weights of ~ 40, 31 and 15 kDa, which would be related to GM-CSF molecules with different degrees of glycosylation from fully glycosylated (40 kDa) to poorly glycosylated (15 kDa). None of the three forms were identified in the negative control that included incubation with a blocking peptide [1:10 in PBS 1% BSA (v/w); GM-CSF peptide orb14376, Biorbyt, St. Francisco, CA, USA], at 4 °C, overnight (Supplementary Figure S3).

### Experimental design

**Figure 7:**
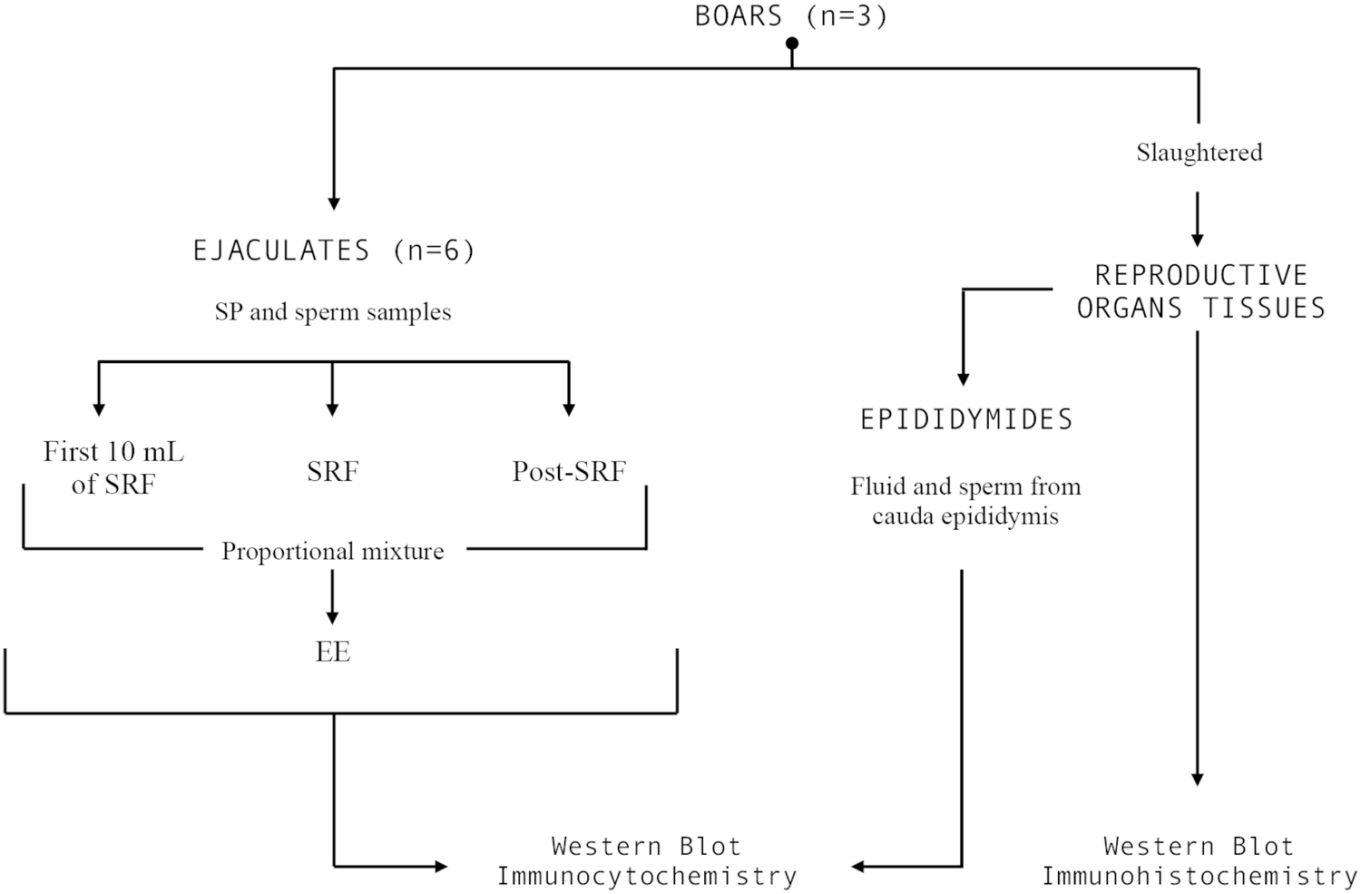
Overview of the experimental design showing how the semen samples (seminal plasma and spermatozoa), epididymal samples (epididymal fluid and spermatozoa) and reproductive organs (testes, epididymis and accessory sexual glands) were obtained and processed.

The sequential order of the experiment carried out is shown in Fig. 7, which describes the number of boars, ejaculates, epididymides and tissue samples evaluated.

### Statistical analysis

Data were analysed using IBM SPSS v24.0 software (IBM Spain, Madrid). In experiment 1, a hierarchical two-step cluster analysis was carried out to identify naturally occurring groups within GM-CSF expression, measured according to fluorescence intensity. Were employed the log-likelihood as distance measure and Schwarz (Bayesian Information Criterion) as clustering criterion, into a sperm data set yielding two clusters having either high or low/absent fluorescent intensity (Supplementary Figure S4). Chi-square test was used for checking differences in the distribution of viable and non-viable spermatozoa between the generated groups. Data of relative content of GM-CSF obtained in WB were logarithmically transformed and putative differences within each sample source (reproductive tissues, ejaculate fractions, SP and epididymal fluid and spermatozoa) were analysed using one-way ANOVA. Post-hoc analyses were performed using the Bonferroni test. Differences with a *p* < 0.05 were considered statistically significant. Data were shown as means ± standard error of the mean (SEM).

## Supplementary information

Supplementary Information.
