# Supplementary material for: Granulocyte-macrophage colony stimulating factor (GM-CSF) is fully expressed in the genital tract, seminal plasma and spermatozoa of male pigs
Source: Sci Rep. 2020 Aug 7;10:13360. doi: 10.1038/s41598-020-70302-9 (PMC7414873; doi:10.1038/s41598-020-70302-9)
Supplement: Supplementary file 1 — Supplementary Information. [file 41598_2020_70302_MOESM1_ESM.pdf]

Granulocyte-Macrophage Colony Stimulating Factor (GM-CSF) is fully expressed in the genital tract, seminal plasma and spermatozoa of male pigs

Lorena Padilla[1,3], Jesús Martínez-Hernández[2,3], Isabel Barranco[1,3,4], Xiomara Lucas[1,3], Luis M. Pastor[2,3], Heriberto Rodriguez-Martínez[5], Jordi Roca[1,3], Inmaculada Parrilla[1,3*]

[1]Department of Medicine and Animal Surgery, Faculty of Veterinary Science, University of Murcia, Murcia 30100, Spain

[2]Department of Cell Biology and Histology, School of Medicine, University of Murcia, Murcia 30100, Spain

[3]IMIB-Arrixaca, Regional Campus of International Excellence, Campus Mare Nostrum, University of Murcia, Murcia 30100, Spain

[4]Biotechnology of Animal and Human Reproduction (TechnoSperm), Department of Biology, Faculty of Sciences, Institute of Food and Agricultural Technology, University of Girona, Girona 17003, Spain

[5]Department of Biomedical and Clinical Sciences (BKV), Linköping University, Linköping, SE 58185, Sweden

* Corresponding author: Inmaculada Parrilla (parrilla@um.es)

Supplemental Information. Six supplementary figures and one supplementary table.

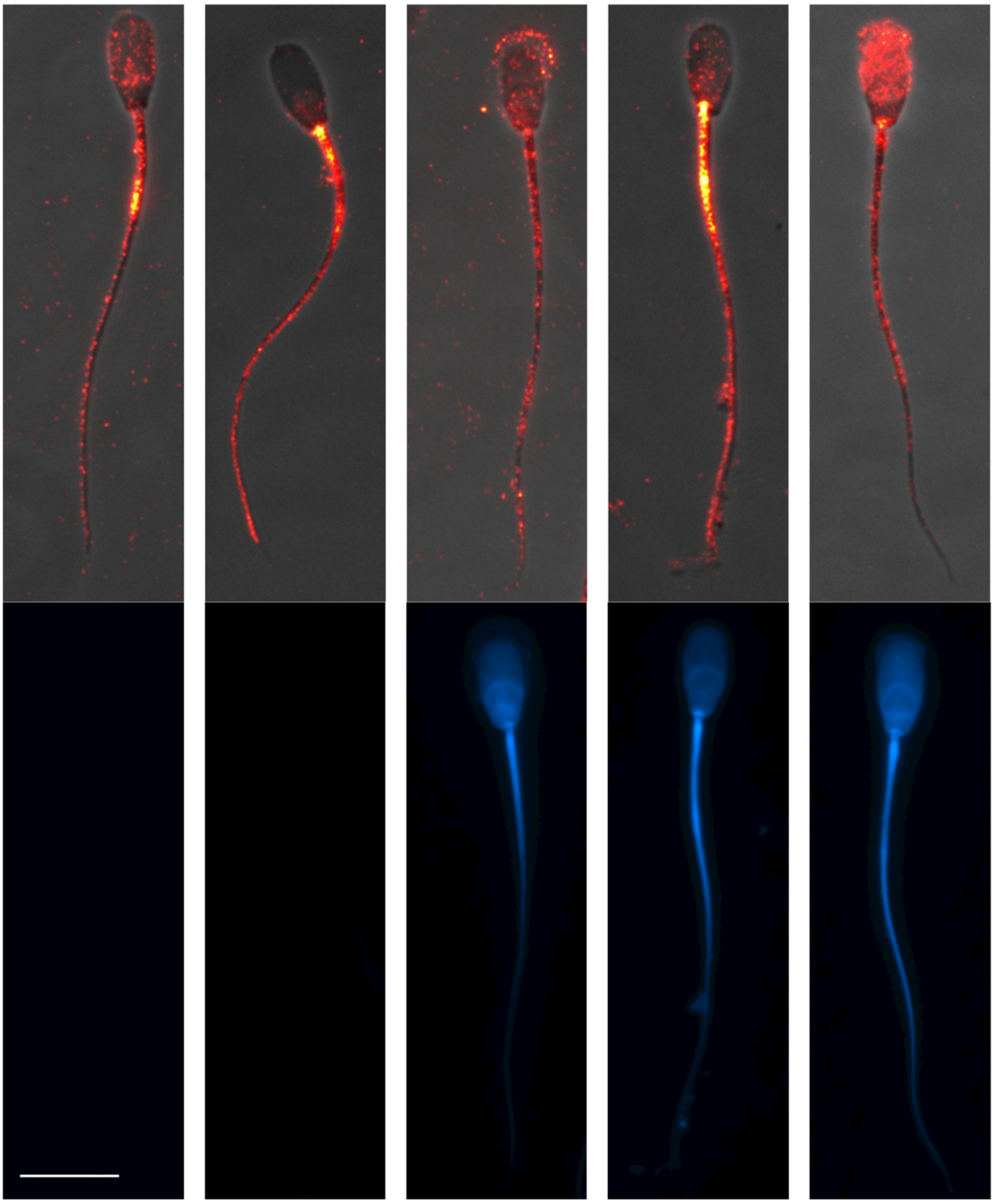

Supplementary Fig S1. Heterogeneous expression of GM-CSF in the pig mature spermatozoa. Sperm samples were incubated with the antibody anti GM-CSF (orb6090, Biorbyt) to identify GM-CSF expression (in red in top images) and with DAPI staining to identify viable from non-viable spermatozoa (DAPI positive in blue in bottom images). Spermatozoa displayed a heterogeneous distribution in fluorescence, more intense in midpiece and sperm flagellum, which is substantially evident in non-viable spermatozoa. Scale bar: 10 μm. Cropped image of immunocytochemistry capture (see Supplemental Information, Fig S5 for full image).

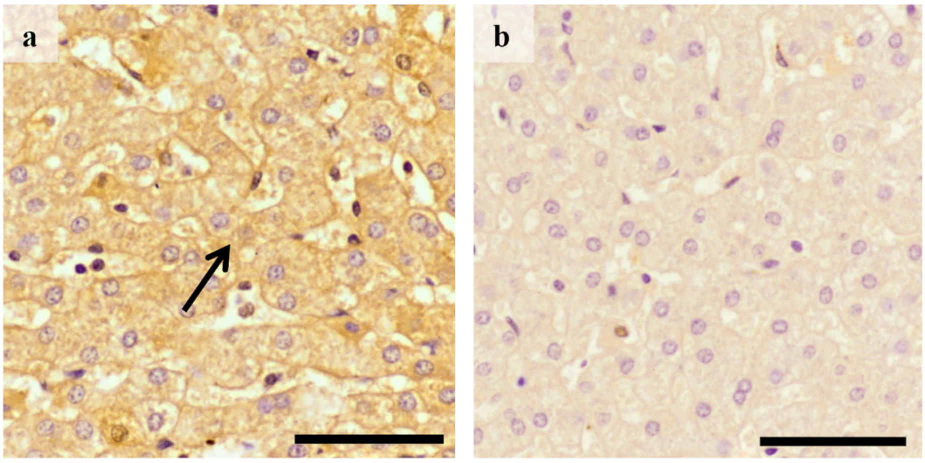

Supplementary Fig S2. Expression of GM-CSF in pig liver. (a) Positive control: Liver tissue incubated with antibody anti GM-CSF (orb6090, Biorbyt) showing expression of GM-CSF in hepatocyte cytoplasm (arrow). (b) Negative control: Liver tissue non-incubated with primary antibody. Scale bar 50μm.

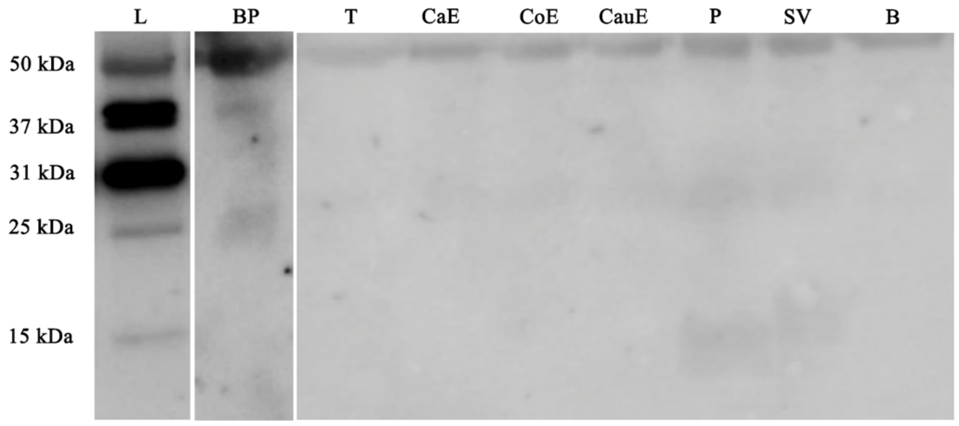

Supplementary Fig S3. Western Blot results of male pig reproductive tissues incubated with blocking peptide. T: testis; CaE: caput epididymis; CoE: corpus epididymis; CauE: cauda epididymis; P: prostate; SV: seminal vesicle; and B: bulbourethral gland. L: Expression of GM-CSF in liver tissue, as control. BP: Liver tissue incubated with blocking peptide. Cropped image of Western blot (see Supplemental Information, Figure S6 for full image).

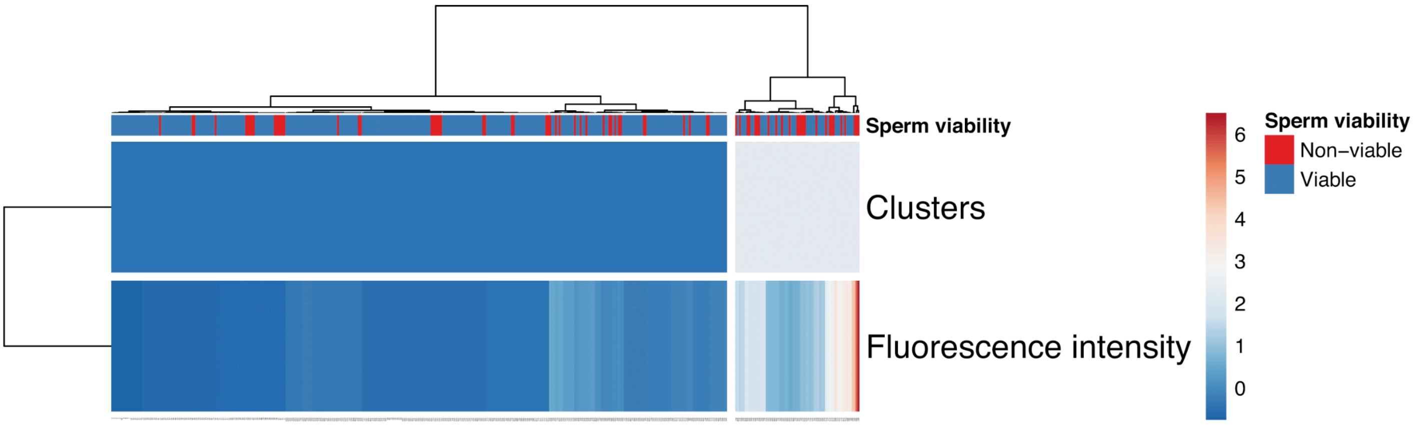

Supplementary Fig S4. Dendrogram. Diagram representing a tree for hierarchical two-step clustering by Euclidean distance and Ward linkage. The color scale indicate the relative expression of GM-CSF fluorescence intensity: red shows higher expression and blue lower expression. Each column represents one spermatozoon. Column annotations indicate the sperm viability, non-viable in red and viable in blue.

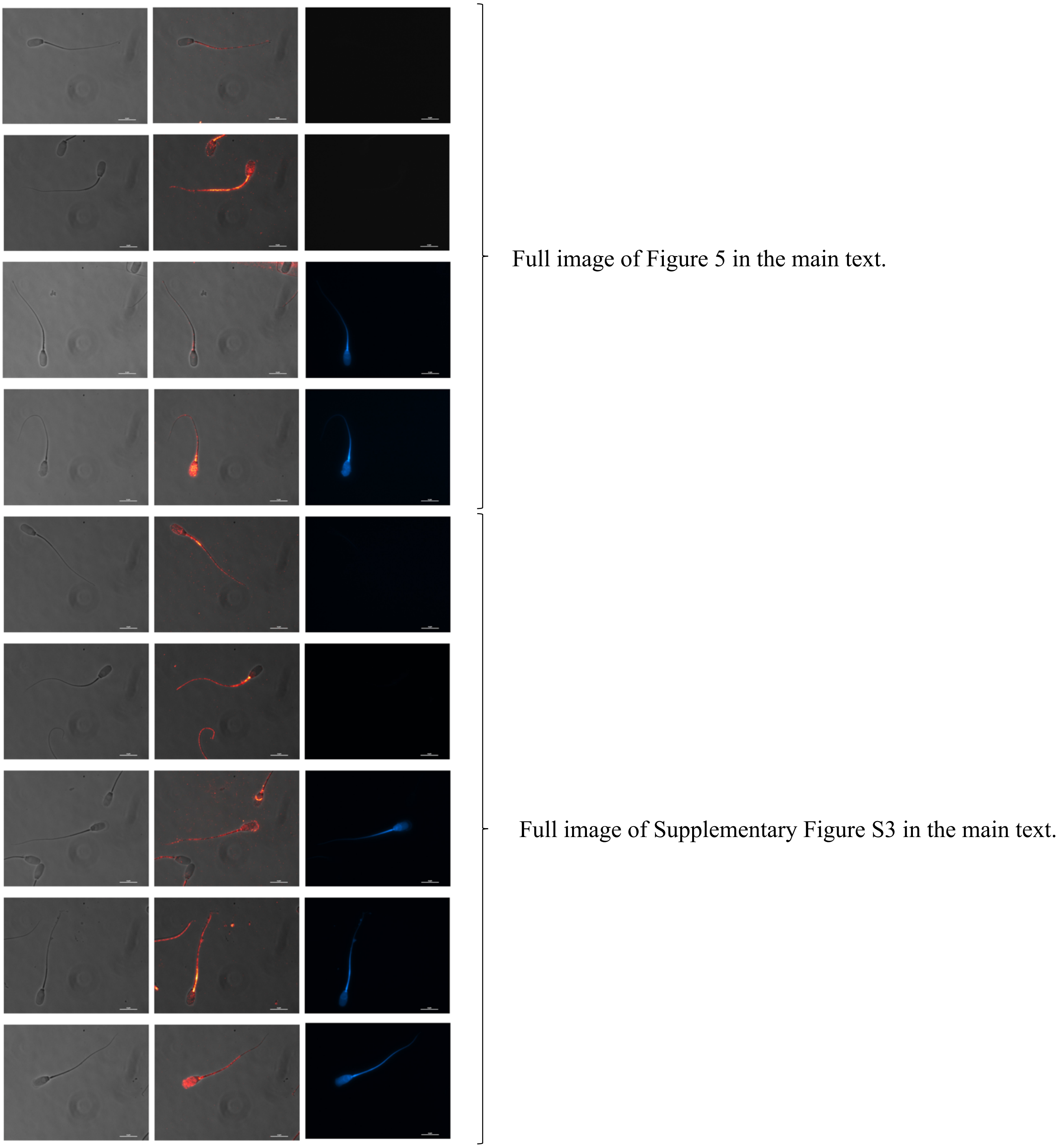

Supplementary Fig S5. Raw data for expression of GM-CSF in the pig mature spermatozoa. Bright-field in left images. Sperm samples were incubated with the antibody anti GM-CSF (orb6090, Biorbyt) to identify GM-CSF expression (composite in red in center images, merging fluorescence and bright-field images) and with DAPI staining to identify viable from non-viable spermatozoa (DAPI positive in blue in right images). The acquisition conditions for bright-field images were 100 ms, 1.00x of gain and high contrast; and for all fluorescence images 150 ms, 1.70x of gain and high contrast. The size of all images was 1280 × 1024 pixels and 32 bit/pixel. The look up table (LUT) used to identify GM-CSF expression was *red hot* and the LUT to identify non-viable spermatozoa, DAPI positive was *cyan hot.* Scale bar 10µm.

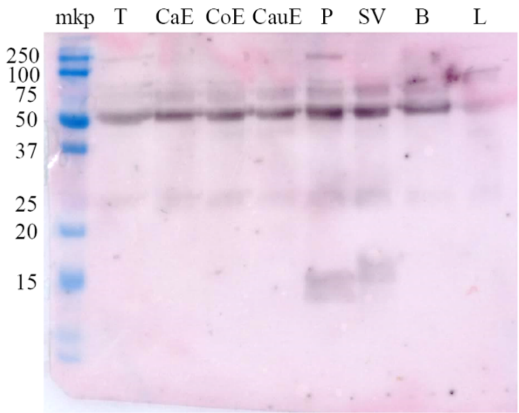

Blocking peptide. Full image of Figure S3

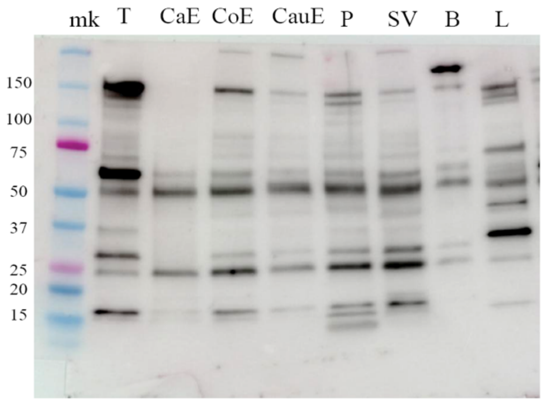

GM-CSF blot in porcine genital tract. 5 min of exposure time. Used in densitometry of 40, 31 and 15 kDa band. **Full image of Figure 7 (a1-4)**

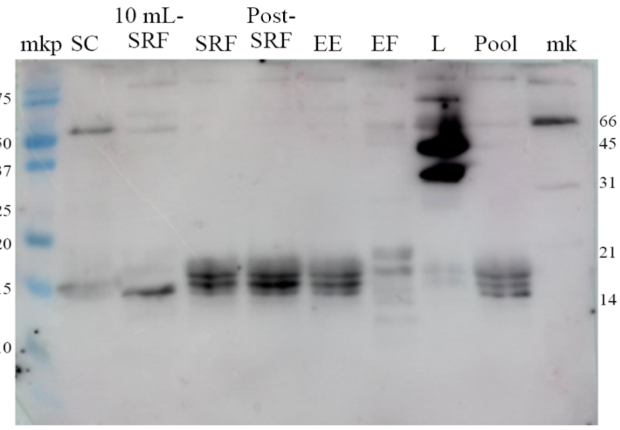

GM-CSF blot in epididymal fluid and seminal plasma from different ejaculated portions. 3 min of exposure time. Used in densitometry of 15 kDa band. **Full image of Figure 7 (b1-2)**

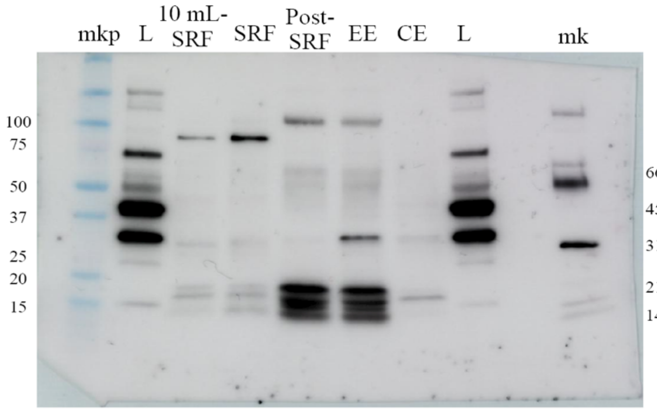

GM-CSF blot in spermatozoa from cauda epididymis and different ejaculated portions. 3 min of exposure time. Used in densitometry of 15 kDa band. **Full image of Figure 7 (c1, c2)**

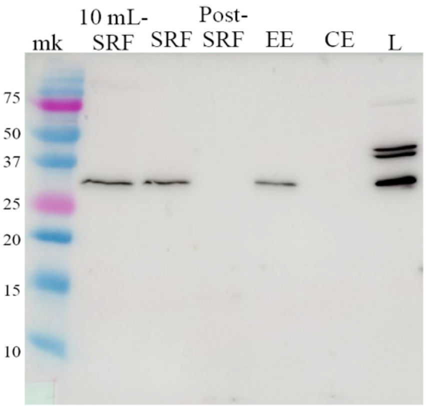

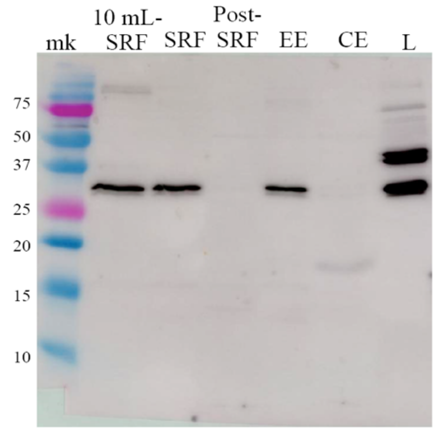

GM-CSF blot in spermatozoa from cauda epididymis and different ejaculated portions. 30 s (left) and 1 min (right) of exposure time. Used in densitometry of 31 kDa band. **Full image of Figure 7 (c1, c3)**

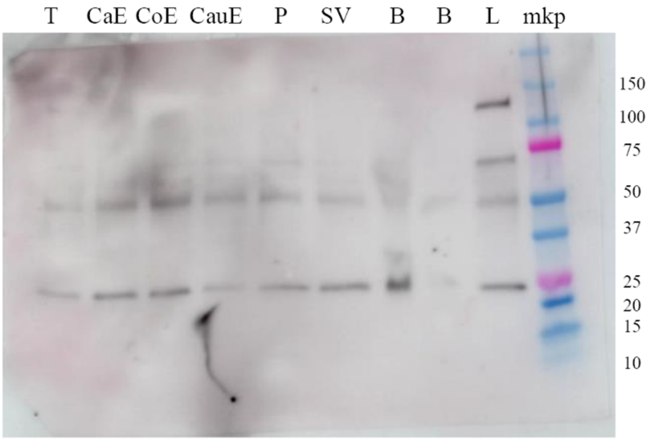

Negative Western blot in porcine genital tract with primary antibody absent. 4 min of exposure time.

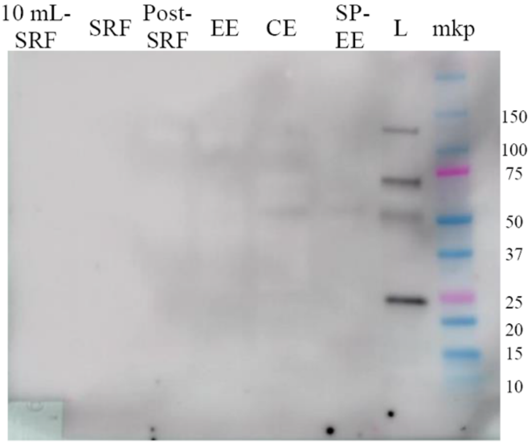

Negative Western blot in spermatozoa from different ejaculated portions and seminal plasma with primary antibody absent. 4 min of exposure time.

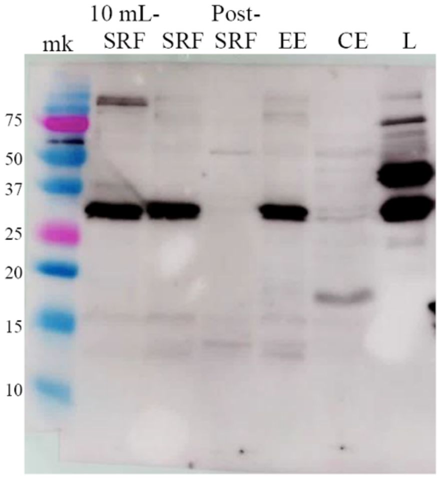

GM-CSF blot in spermatozoa from cauda epididymis and different ejaculated portions. 2 min of exposure time. Not used in densitometry.

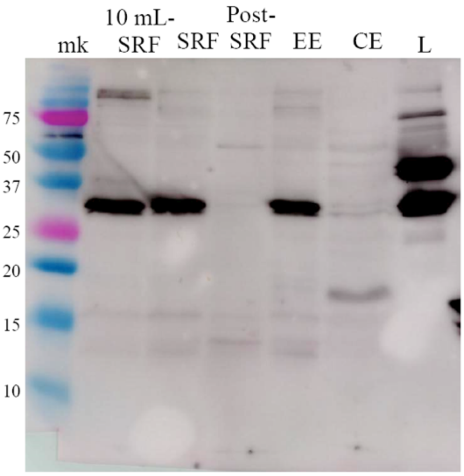

GM-CSF blot in spermatozoa from cauda epididymis and different ejaculated portions. 3 min of exposure time. Not used in densitometry.

mkp: Precision Plus Protein Dual Colour Standards. Catalog Number #1610374
mk: SDS-PAGE Molecular Weight Standards. Broad Range. Catalog Number 161-0317
SC: HeLa Whole Cell Lysate: Positive control SC-2200 Santa Cruz Biotechnology

Supplementary Fig S6. Raw data for Western blot results. T: testis; CaE: caput epididymis; CoE: corpus epididymis; CauE: cauda epididymis; P: prostate; SV: seminal vesicle; B: bulbourethral gland; epididymal fluid: EF; SP from the different ejaculate portions: first 10 mL of the SRF (10 mL-SRF), rest of SRF, post-SRF, entire ejaculate (EE); mature spermatozoa from cauda epididymis (CE), 10 mL-SRF, rest of SRF, post-SRF and EE. L: expression in liver tissue, as control. Western blot densitometry was analysed using different exposure times in blots, avoiding satured Western blot.

Supplementary Table S1. Quality parameters (mean percentage ± SEM) of pig mature spermatozoa from the cauda epididymis and ejaculate fractions, namely first 10 mL of the sperm-rich fraction [SRF], rest of SFR, post-SRF and entire ejaculate [EE] collected to male pigs used in study.

Sperm parameters (%)	Source of mature spermatozoa				
		Ejaculate			
	Cauda epididymis	First 10 mL-SRF	Rest of SRF	Post-SRF	EE
Total motility[1]	60.3±2.4	82.0±2.5	80.7±5.8	71.7±4.0	83.0±3.8
Progressive motility[1]	37.0±2.6	40.7±3.2	42.7±9.8	40.7±11.6	49.0±8.7
Viability[2]	94.5±0.5	84.5±6.4	87.5±2.1	77.9±6.4	88.3±1.6

[1]Sperm motility was evaluated using CASA (Proiser R+D, Paterna, Spain). Total motility was recorded as the percentage of total motile spermatozoa (average path velocity ≥ 20 µm/s); progressive motility was the proportion of motile spermatozoa showing rapid and progressive movement (straight line velocity ≥ 40 µm/sec).

[2]Sperm viability was assessed by flow cytometry (BD FACS Canto II flow cytometer; Becton Dickinson & Company, Franklin Lakes, NJ, USA). Triple stain was performed by the association of Hoechst 33342 (H-42) with Propidium Iodide (PI) and peanut agglutinin conjugated to FITC (PNA-FITC). Viable spermatozoa were those H-42 positive exhibiting intact plasma and acrosome membranes (PI negative and PNA-FITC negative) and were reported as percentages.
